# Targeting Conventional Dendritic Cells to Fine-Tune Antibody Responses

**DOI:** 10.3389/fimmu.2019.01529

**Published:** 2019-07-04

**Authors:** Demo Yemane Tesfaye, Arnar Gudjonsson, Bjarne Bogen, Even Fossum

**Affiliations:** ^1^K. G. Jebsen Center for Research on Influenza Vaccines, Oslo University Hospital, University of Oslo, Oslo, Norway; ^2^Department of Immunology and Transfusion Medicine, Oslo University Hospital, Oslo, Norway; ^3^Institute of Clinical Medicine, Oslo University Hospital, University of Oslo, Oslo, Norway

**Keywords:** dendritic cell (DC), DC subtypes, vaccination, targeting, antibody, Th1 & Th2

## Abstract

Dendritic cells (DCs) facilitate cross talk between the innate and adaptive immune system. They sense and phagocytose invading pathogens, and are not only capable of activating naïve T cells, but can also determine the polarization of T cell responses into different effector subtypes. Polarized T cells in turn have a crucial role in antibody class switching and affinity maturation, and consequently the quality of the resulting humoral immunity. Targeting vaccines to DCs thus provides a great deal of opportunities for influencing the humoral immune responses, by fine-tuning the T cell response as well as regulating antigen availability for B cells. In this review we aim to outline how different DC targeted vaccination strategies can be utilized to induce a desired humoral immune response. A range of factors, including route of vaccine administration, use of adjuvants, choice of DC subset and surface receptor to target have been reported to influence the resulting immune response and will be reviewed herein. Finally, we will discuss opportunities for designing improved vaccines and challenges with translating this knowledge into clinical or veterinary medicine.

## Introduction

Conventional dendritic cells (cDCs) are divided into two sub-populations, cDC1s and cDC2s, based on ontogenic and functional differences [reviewed in (1, 2)]. Both populations are derived from pre-cDCs that develop in the bone barrow, before migrating to secondary lymphoid or peripheral tissue where the final differentiation into cDC1s and cDC2s occurs (3–5). Differentiation into cDC1s is dependent on the transcription factors IRF8 (6) and Id2 (7), and to a lesser extent BATF3 (8, 9), while cDC2 differentiation is dependent on IRF4 (10, 11). Both cDC1s and cDC2s can present antigen-derived peptides on MHC-II to CD4^+^ T cells, although studies have reported that cDC2s are more efficient at this process (12, 13). In contrast, only cDC1s efficiently cross-present antigens to CD8^+^ T cells in mice (13–15).

Due to their crucial role in the induction of T cell responses, delivery of antigen to DCs has been extensively evaluated in various cancer and infectious disease models [reviewed in (16)]. However, targeting antigen to DCs can also enhance antibody responses. Indeed, early studies where avidin was conjugated to biotinylated anti-MHC-II antibodies resulted in improved induction of anti-avidin antibodies in the absence of adjuvant (17). However, since MHC-II is also expressed on B cells and other antigen presenting cells (APC), it was unclear to what extent DC targeting contributed to the improved antibody responses. Wang and colleagues later observed that targeting the pan-DC marker CD11c also resulted in enhanced antibody responses, highlighting DCs as an attractive target for enhancing humoral responses (18). Here, we review the current literature on how delivering antigens to cDCs can be utilized to enhance both the polarization and the magnitude of the humoral response, and discuss how different immunization strategies can be utilized to fine-tune the antibody responses.

## Antibody Polarization as a Function of DC Subtype

**Figure 1:**
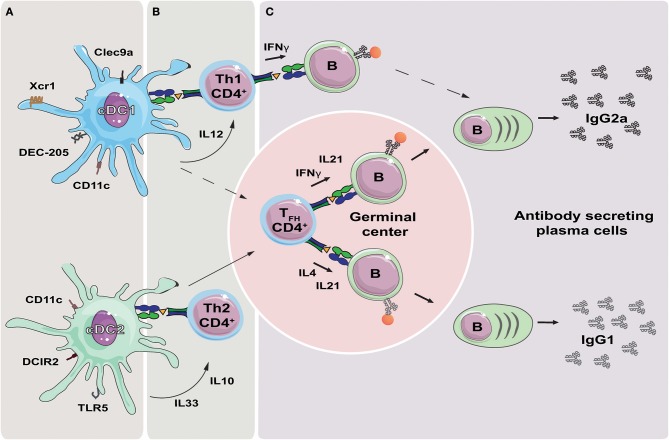
Targeting DC subsets to influence the polarization of the antibody response. **(A)** Targeting the receptors Xcr1, Clec9a and DEC-205 can be used to deliver antigen to cDC1s, while targeting DCIR2 and TLR5 will deliver antigen to cDC2s. Targeting CD11c will deliver antigen to both DC subsets. **(B)** Delivery of antigen to cDC1s or cDC2s will result in presentation of antigen derived peptides on MHC-II. Secretion of cytokines such as IL-12 by cDC1s, or IL10 and IL33 by cDC2s, can initiate polarization of the T helper cell responses in direction of Th1 or Th2, respectively. In addition, both cDC1s and cDC2s have been reported to induce T_FH_ cells, although cDC2s are likely more important in this respect. **(C)** T_FH_ cells migrate to germinal center where they regulate isotype switching of antigen specific B cells through secretion of IFNγ (IgG2a) or IL4 (IgG1). Th1 may also contribute to isotype switching through secretion of IFNγ, while it is less clear if Th2 cells contribute to IgG1 switching by secretion of IL4. T_FH_ cells further regulate affinity maturation of the antigen specific B cells, and secrete IL-21 resulting in the formation of plasma cells and secretion of high affinity antibodies.

DCs are effective in priming naïve CD4^+^ T cells into functionally distinct effector T helper (Th) cells, with different subsets of DCs dictating differential effector T cell commitment (19, 20). cDC1s promote development of T helper 1 (Th1) cells through secretion of IL-12 (21), while cDC2s secrete IL-10 and IL-33 and drive Th2 responses (22) (Figure 1). In addition, cDC2s are important for efficient induction of T follicular helper (T_FH_) cells (23, 24). It is less clear to what extent cDC1s influence T_FH_ induction, but they have been reported to do so under inflammatory conditions (25). Also, studies have shown that IL-12 secreting DCs can promote T_FH_ induction in humans (26, 27). The different T helper subtypes, through secretion of different cytokines, will in turn influence the magnitude and nature of humoral immune responses mounted by B cells (28, 29). For instance, secretion of IL-21 by T_FH_ cells induces differentiation of naïve and memory B cells into antibody secreting plasma cells (26, 30, 31). Furthermore, the characteristic Th1 cytokine IFN-γ promotes induction of IgG2a class switching, while the Th2 associated IL-4 drives class switching toward IgG1, in mice (Figure 1) (32, 33). Recent studies have identified subtypes of T_FH_, now often referred to as Tfh1 and Tfh2, as the source of IFN-γ and IL-4, respectively (34). However, induction of IgG2c has been observed in mice deficient of T_FH_ cells, suggesting that Th1 cells may be sufficient for class switching (35).

Mice have four subtypes of IgG: IgG1, IgG2b, IgG3, and depending on the strain, IgG2a in BALB/C or IgG2c in C57BL/6 (31, 36). The fragment crystallizable (Fc) region of the different IgG subtypes binds to specific Fc-receptors (FcγRs) and modulate subsequent effector cell functions (37). In mice there are three activating Fcγ-receptors (FcγRI, FcγRIII, FcγRIV) and one inhibitory Fcγ-receptor (FcγRIIB) (38). IgG2a and IgG2b are reported to have higher affinity for the activating Fcγ-receptors which result in pro-inflammatory responses (39) and antibody-dependent cytotoxicity (ADCC) (40). In contrast, IgG1 has higher affinity for the inhibitory FcγRIIB, and therefore contributes to dampening the inflammatory response (41).

Several recent studies have observed that antibodies directed against certain viral antigens heavily rely on Fc-mediated effector functions to provide protection. For instance, broadly neutralizing antibodies against HIV gp120 are more efficient as IgG2c subtype compared to IgG1 (42). Similar observations have been made with broadly neutralizing antibodies against the stem-region (43) or head-region (44) of influenza hemagglutinin (HA), and the highly conserved influenza M2e antigen (45, 46). In humans, IgG3 is one of the subtypes with high affinity for activating Fcγ-receptors, and consequently mediates strong effector functions (38). Interestingly, a large phase 3 HIV vaccine trial in Thailand observed a correlation between IgG3 titers and partial protection against HIV infection (47).

Together, these studies demonstrate how DC subsets, through T cell polarization, can influence antibody subclass polarization and consequently vaccine efficacy. They also raise the intriguing possibility of delivering different antigens to cDC1s or cDC2s at the same time, and eliciting different polarized immune responses to different antigens. Such approaches could be of value in the development of vaccines against more complex microorganisms, such as *Mycobacterium tuberculosis*, where conventional vaccine technology has yet to produce an efficient vaccine.

## Targeting Different DC Receptors Impacts Antibody Polarization

The propensity of the different DC subtypes to elicit different arms of the adaptive immune response creates opportunities for fine-tuning of vaccine induced immune responses. In accordance with the described role of DC subsets, targeting influenza HA to cDC1s by fusion to the chemokine Xcl1, the ligand for the cDC1 restricted receptor Xcr1 (48, 49), induces Th1 cells and antibody responses dominated by the IgG2a subclass (50–52). Similarly, targeting the model antigen ovalbumin (OVA) to the C-type lectin DEC-205 on cDC1s has also been shown to increase IgG2a and IgG2b responses compared non-targeted controls (53). However, targeting the cDC1 restricted C-type lectin Clec9a has been reported to induce more of a mixed IgG1/IgG2a response, suggesting that the choice of the targeted receptor also influences the antibody polarization, and not just the targeted DC subset. The enhanced induction of IgG1 may be related to the increased T_FH_ induction observed when targeting antigen to Clec9a (54).

Delivering antigen to surface receptors expressed on cDC2s, such as toll like receptor 5 (TLR5) or DC-inhibitory factor 2 (DCIR2), results in antibody responses of the IgG1 subclass (52, 55). However, the exact mechanism of Th differentiation and whether or not germinal center (GC) formation is enhanced during DCIR2 targeting is less clear. For instance, Chappell and colleagues reported that during DCIR2 targeted vaccination, Tfh differentiation is achieved indirectly through activation of extrafollicular B cells, which ultimately results in an IgG1 response (55). In this study, it was reported that DCIR2 targeting failed to induce germinal center formation (55). However, Shin et al. (24, 56) have reported that DCIR2 targeted vaccination in the presence of poly(I:C) or LPS is capable of efficiently inducing GC formation and T_FH_ differentiation.

In a comparative study, Do and colleagues targeted antigen from *Yersinia pestis* to DEC-205 or DCIR2, and observed distinct cytokine profile associated with Th1 or Th2 polarization, respectively (57). Consistent with these observations, targeting CIRE and FIRE (C-type lectin family receptors on murine cDC2s) using rat anti-CIRE and anti-FIRE antibodies results in enhanced antibody production of the IgG1 subtype compared to rat anti-DEC-205 antibodies (58). In this study, the antibody profile obtained from targeting cDC2s was limited to IgG1, while DEC-205 targeting induced IgG2a, and IgG3 responses mixed with IgG1 (58). Consequently, targeting cDC1 or cDC2 predominantly polarize the antibody response toward IgG2a/IgG2c or IgG1, respectively.

Targeting antigen to surface receptors expressed on both cDC1s and cDC2s, such as CD11c or MHC-II, have yielded antibody responses dominated by the IgG1 subclass (18, 52, 59). One potential explanation for this observation is that cDC2s constitute 80–90% of the cDCs in secondary lymphoid organs (60, 61), and would therefore be more likely to obtain the antigen. Interestingly, fusing influenza HA to the chemokine CCL3, a ligand for CCR3 and CCR5 expressed on both cDC1s and cDC2s, resulted in a more mixed IgG1 and IgG2a response (62, 63). Adding to the complexity, vaccine delivery site may also contribute to the polarization of the antibody response. For example, we recently observed that CCL3-HA induced a significantly more Th1 polarized antibodies response after intramuscular DNA immunization compared to intradermal DNA immunization (63), despite reports of similar ratio of cDC1s to cDC2s in the two tissues (15, 64).

It should be noted that adjuvants also influence immune responses during cDC1 and cDC2 targeted vaccinations. At steady state, immature DCs tend to induce tolerogenic responses [reviewed in (65)] while presence of adjuvants stimulates upregulation of maturation markers and abrogates tolerance induction (66). During DEC-205 targeted cDC1 vaccinations, co-administration of adjuvants was important in inducing immune responses (58). However, targeting Clec9a, another receptor on cDC1, is reported to be immunogenic even in the absence of adjuvants (54). Furthermore, presence of adjuvants influences the efficiency of DCIR2 targeting on formation of GC reactions and T_FH_ polarization (24, 55, 56). Of note here is that adjuvants may act on other immune cells than cDCs to dictate the immune outcome. For instance, a synthetic hemozoin adjuvant has recently been shown to interact directly with B cells and enhance IgG2c class switching (67). In this regard, addition of Th1 polarizing adjuvant can boost the induction of Th1 associated IgG subclasses even when delivering antigens to cDC2s. For instance, targeting antigen to DCIR2 in the presence of anti-CD40 and polyI:C increased titers of both the Th1 (IgG2c or IgG2a) and the Th2 (IgG1) subclasses (57, 68). All in all, the variation in choice of vaccine design coupled with differences in adjuvants used, makes the direct comparison of DC targeted vaccination from different studies difficult. Therefore, comparative investigations of antibody polarization profiles after targeting DCs using similar vaccine platforms is crucial.

## Magnitude of the Antibody Response

While the ability to impact the polarization of the antibody response is important, the magnitude and the affinity of the antibodies will ultimately determine whether the vaccine is protective or not. With regards to achieving good antibody responses, Clec9a has been reported to be a promising targeting candidate in mice using the model antigen OVA as well as the influenza antigen M2e and the hand, foot and mouth disease-causing enterovirus 71 antigen SP70 (54, 69). This effect of Clec9a targeting has in part been attributed to efficient induction of T_FH_ cells (54, 70). Targeting antigen to the pan-DC marker CD11c has also resulted in high antibody titers as well as improved germinal center responses (18, 71). While CD11c, and especially Clec9a, are predominantly expressed on cDCs, there are other markers worth mentioning that are not as DC specific. For instance, targeting influenza HA to MHC-II or NP and OVA to CD180 have resulted in strong antibody responses (59, 71–73). While both of these markers are expressed on DCs, they are not DC specific. Indeed, it is the proliferation and differentiation of the B cells that has been shown to be of importance in inducing antibody responses upon targeting CD180 (73). Similarly, Andersen et al recently used adoptively transferred B cells to show that targeting MHC-II exclusively on B cells, in the absence of DCs, is sufficient to increase antibody production (74).

## Modulating Antigen Availability Kinetics to Increase Antibody Responses

Recently, the augmentation of vaccine induced antibody responses through modulation of antigen availability kinetics has emerged as a promising strategy in rational vaccine design. Encouraging findings in mice using HIV Env trimers or HIV gp120 as antigen have shown that prolonged antigen release gives better antibody responses when compared to bolus vaccination—an effect that may be further improved by exponentially increasing the immunization dose (75, 76). Studies in non-human primates have shown similar effects (77, 78), demonstrating the translational potential of this immunization strategy. One possible explanation for the improved antibody responses may be that these vaccination strategies more closely mimic antigen availability kinetics of natural infections. Further beneficial effects of prolonged antigen availability applied to vaccinations are reviewed in more detail elsewhere (79).

The objective of DC targeted vaccine strategies is the manipulation of antigen delivery to DCs, which in turn directly affects antigen availability for B cells. This latter aspect presents opportunities that perhaps have not been fully appreciated in the field. Targeting strategies could be designed to optimize the antigen availability kinetics for B cells in order to augment the antibody response, aiming to achieve the same effects as those seen with prolonged antigen release in the above-mentioned studies. For instance, we have observed that when targeting hemagglutinin influenza HA to the Xcr1 receptor on cDC1s in a manner that does not result in receptor activation and internalization, the ensuing GC and antibody responses were greater than when the vaccine was actively endocytosed (51, 80). A strength in our approach is that we directly compare targeting to the same receptor on cDC1s in a manner that either induces endocytosis or not, while keeping all other variables constant. Similarly, the strong antibody responses observed when targeting Clec9a are suggested to be due to high specificity of Clec9a expression resulting in long half-life of the vaccine molecule in serum after immunization, thus prolonging availability of the antigen to B cells (54). Notably, a DEC-205 targeted vaccine in the same study had a shorter half-life and was less efficient in inducing antibody responses (54). These effects were obtained in the absence of adjuvants, supporting the notion that they were purely due to the nature of the targeting.

Finally, the choice of delivery method may affect the antigen availability kinetics. For instance, DNA vaccination may result in more optimal antigen availability kinetics than bolus protein vaccination. The antigen will be produced and released over a prolonged time, more resembling a natural infection and delivering fresh and intact antigen to the GC response after its initiation. Nevertheless, DC targeting strategies aiming to achieve good antibody responses should be designed with the B cell antigen availability kinetics in mind, whether they are based on DNA or protein immunization.

From these observations, we can summarize that targeted vaccines that are less efficiently taken up and degraded by DCs are more likely to be potent in inducing antibody responses. Such vaccines may result in more antigen being available in the lymph nodes for a longer period of time, potentiating the GC reaction. Additionally, if the vaccine molecule stays on the DC surface, it might interact with and recruit B cells entering the draining lymph node through high endothelial venules in the form of a DC-B cell synapse (81).

## Translation to Clinical Medicine

Translating vaccine strategies aimed at delivering antigen to DCs to humans present a number of challenges. First, DC subsets in mice and humans have until recently been defined by different surface markers, making it challenging to compare observations between the different species. However, with the discovery of more conserved markers for cDC1s such as XCR1 (82–84), CLEC9A (85–87), and for cDC2s such as SIRP1a (60), more accurate strategies for defining DC populations in both man and mouse have become available (60). Second, expression of specific surface markers may vary between species. For example, DEC-205 has been reported to have a broader expression pattern in humans compared to mice (88), adding uncertainty to how pre-clinical data from mice will translate to humans. Third, it is unclear to what extent the functional separation of cDC1s and cDC2s seen in mice is conserved in humans. For instance, cross-presentation to CD8^+^ T cells and induction of Th1 cells has been associated with cDC1s in mice. In humans, however, both cDC1s and cDC2s have been reported to cross-present antigen (89), secrete IL-12 and promote Th1 polarization (90, 91). Nevertheless, clinical trials have demonstrated that targeting the tumor antigen NY-ESO-1 to DEC-205 induced antigen specific antibodies (92). The antibody responses were dominated by IgG1, which is functionally more similar to the IgG2a subclass in mice (38).

While delivering antigen to DEC-205 is the only DC targeting strategy to have entered into clinical trials so far, studies in other animal models have been reported. Immunization of Macaques with a rat antibody specific for Macaque CLEC9A resulted in strong anti-rat IgG antibody responses in the absence of adjuvant (93). In pigs, targeting the influenza antigen M2e to cDC1s by fusion to porcine XCL1 enhanced antibody responses in both naive and sero-positive animals (94). Also in pigs, targeting the influenza antigen nucleoprotein (NP) to CD11c induced strong antibody responses, although the responses were not enhanced compared to non-targeted controls (95). With more DC targeting strategies being evaluated in multiple species, we should obtain a better understanding of how characteristics like Th1/Th2 polarization and magnitude of the antibody response translate from mouse studies.

## Concluding Remarks

Delivering antigens to cDCs has garnered much attention as a method for enhancing CD8^+^ T cell responses. We strongly believe that the potential to influence CD4^+^ T cell polarization, and consequently antibody class switching, are of equal importance. Especially when considering the observations that IgG-subclass and Fc-mediated effector functions strongly influence the protective ability for broadly neutralizing antibodies against influenza and HIV. When also considering the ability to actively manipulate the magnitude and quality of the antibody responses, DC targeting will be a valuable tool for further vaccine development.

## Author Contributions

All authors listed have made a substantial, direct and intellectual contribution to the work, and approved it for publication.

### Conflict of Interest Statement

The TTO office of Oslo University and Oslo University Hospital has filed several patents on the use of Vaccibodies, on which BB is an inventor. BB is head of the scientific advisory board of the Vaccibody Company and holds shares in the company. The remaining authors declare that the research was conducted in the absence of any commercial or financial relationships that could be construed as a potential conflict of interest.
